# Skin Diseases and Syphilis

**Published:** 1897-09

**Authors:** 


### *SELECTIONS AND ABSTRACTS.*

---

### SKIN DISEASES AND SYPHILIS.

EDITED BY M. B. HUTCHINS, M.D.

### "COLLES'S LAW." AN APPARENT EXCEPTION.

Dr. Wm. T. Corlett, of Cleveland, Ohio, reports in the June (1897) *Journal of Cutaneous and Genito-Urinary Diseases*, a case which seems a partial refutation of this "law."

Colles had never seen a mother who nursed her syphilitic infant infected by it, even though she had borne the child to a syphilitic father without evident syphilis in herself.

The idea is that the mother becomes immune through bearing the child. Sufficient cases, however, have occurred to show that immunity does not always follow.

L. Merz (abstract in Ann. de Derm. et de Syph., 1890, p. 532) had a perfect case. Ogilvie (London *Lancet*, February 1, 1896) had collected twenty cases contradictory to "Colles's law," and Sturges and others have had a like experience.

Dr. Corlett's case was that of a woman who had a congenitally syphilitic child, with mucous patches in the mouth. Two weeks after these appeared the mother developed a lesion on the left breast, two inches from the nipple, which presented the symptoms of an extra-genital chancre. Axillary and post-cervical adenopathy followed. The doctor was not able to follow the case further, though he believes that the mother was infected from her syphilitic child. He well says that an absence of immunity is seen, exceptionally, in cases of other diseases where we would expect it to be present.

(To prove an actual exception to the law it is necessary to eliminate all chances of early acquired (and not congenital) syphilis. A lesion of epithelium and the poison of syphilis brought together are all-sufficient to the acquirement of the disease.)

### FATTY SEBORRHEA AND ALOPECIA AREATA.

*The Journal of Cutaneous and Genito-Urinary Diseases* for June, 1897, has a reference to a paper of R. Saboraud (An. de l'Inst. Pasteur, February 25, 1897). He says that the germ of oily seborrhea and of these cases of sudden, smooth, loss of hair is identical. The germs are found in a "cocoon" in the hair-follicle between its mouth and the mouth of its sebaceous gland. The presence of this micro-organism cocoon causes the fall of hair. Sulphur preparations are the most useful remedies in either disease.

Saboraud produced characteristic bald areas, with pure cultures, on the "calf, rabbit and guinea-pig."

(If further research and experiment confirm Saboraud's conclusions, the present methods of treatment of oily seborrhea will be intelligently modified and former ideas regarding alopecia areata and its treatment be revolutionized.)

---

### THE TREATMENT OF CUTANEOUS CANCER.

Dr. A. R. Robinson, the well known authority, has a paper upon this subject in the New York *Polyclinic Journal* for July 15, 1897. His views remain the same as those which he has expressed in previous papers. He rightly condemns all mild methods of treatment, as they but stimulate the disease to greater activity. He also rightly considers electricity effective only in cases where it is used as a caustic. Erysipelas toxins are valueless. The knife is recommended only where much tissue can be removed. Caustic potash, and arsenious acid, in the form of Marsden's paste, are his chief reliance.

He expects these agents to destroy all of the visible disease, then next to it a number of cells beyond the visible border; and, finally, the few cells which lie still further away by the inflammatory action of the caustic. He claims for caustics a greater destruction of disease and less deformity than possible with the knife. (Certain regions can best be treated with caustic applications, and they have a wide utility, but they may fail, even when used properly, as Robinson insists they must be, and the question comes down to the axiom "get all of the disease." One must do this no matter what is used, and results alone can show, when two or three years old, that one method must supersede all others.)

---

### GENERAL, SECONDARY CARCINOMA OF THE BONES.

Dr. J. Ritchie reports this case in the Edinburgh *Medical Journal*, September, 1896. Seven years after delivery a woman of forty years was operated upon, by Annandale, for carcinoma of the right breast and the axillary glands. There was no local recurrence, but six years and eight months later sudden and severe pains began in the back and limbs, which were later found to be the beginning of a condition resembling osteomalacia of the bones of the thorax and pelvis. Shortly before her death, at fifty-six, a slight movement in bed produced a fracture of the fourth rib near the cartilage. Carcinoma cells were demonstrated in the bones, by the microscope, which had produced this pseudo-osteomalacia. Such cases show that carcinoma cells can remain in a bone or the bones for years without the production of noticeable symptoms, and even outward change of form is frequently unrecognizable. These considerations render the diagnosis of single cases difficult.—(*Translated*) *Centralb. f. Chirurg*, May 29, 1897.

---

### THE TREATMENT OF PRURITUS PUDENDORUM AND PRURITUS ANI.

A. Haidenhain, *Berlin. Klin. Wochenschr*, 1897, No. 2, has used the principle of the good effect of a high degree of heat on itching parts by applying compresses wet with a mixture of a heaping tablespoonful of tannic acid to the litre of hot water. He says every pruritus is curable in this way. For pruritus pudendorum he makes antiseptic vaginal injections, lysol (a German antiseptic), lukewarm water, bichloride solution, in order. Then he places gauze pledgets wet with the tannin solution between the labia majora. He attributes the good effect of the tannin to its disinfectant properties. It ruins the clothing, though.—(*Translated*) *Centralb. f. Chirurg.*, May 22, 1897.

Dr. M. S. Alexander (in *New York Medical Journal*, January, quoted by *Jour. A. M. A.*, August 21, 1897) claims to have cured an old case of pruritus vulvæ with formalin (strength not given in quotation), and claims generally good results for it in "skin troubles." (The formalin should first be used in weak solution, gradually approaching the proper strength, even if the proper strength should be the full forty per cent.)

---

A great many authors who report upon the "value" of new drugs entirely nullify the worth of such reports by claiming that the whole good result comes from the new remedy, while their communication states that other drugs with much the same action were used at the same time. Such reports are absolutely worthless.

---

### SUCCESSFUL TREATMENT OF SARCOMA BY ELECTROLYSIS AND CATAPHORESIS, COMBINED WITH THE INTERNAL USE OF DONOVAN'S SOLUTION.

This paper was read by Dr. J. McF. Gaston before the American Surgical Association, May 6, 1897, and has since been published in pamphlet form. At the time Dr. Gaston began his treatment of the case he was not aware of previously reported cases by Massey.

The small round-celled sarcoma was situated in the hypogastric region of a boy of twelve years, and was six by seven inches. It was inoperable.

He gave the boy, internally, about eight drops of Donovan's solution t. i. d. with syr. trifol. comp. He attached the needle to the positive pole of a twelve-cell Waite & Bartlett battery, inserting the needle in the right side of the tumor—the sponge on the negative pole being placed over the left of the tumor. At first the current from six cells was used, for five minutes. (No milliammeter was used. The actual, working current was perhaps one-half to two milliamperes.) He finally got up to ten minutes' treatment daily, later alternate days and finally only every third day. Punctures were about half an inch apart, going around the border of the tumor. Meanwhile he had begun using the needle to the negative pole and cataphoresis with Donovan's solution from the positive. He had to judge of the current's strength by the effect. He succeeded, in four months, in reducing the tumor to near a fourth of its original size. The internal treatment had been kept up, and the diet regulated. The doctor's plan of treatment and its execution were original with him, though Dr. G. Betton Massey had done the same kind of work.

It is to be regretted that Doctor Gaston had no means of accurately measuring the current actually used. As to the results, he is to be congratulated, even though that result is not a cure.

The sarcomata have often shown themselves amenable to treatments, to the extent of being reduced, and a few even cured, thereby.

Hypodermatic injections of arsenic, Coley's treatment and many other things have been beneficial. A few, especially of the skin, have spontaneously subsided.

(The sarcomata are mesoblastic tumors. So is a mole. The carcinomata are of epi- or hypo-blastic origin. A wart is more of an epiblastic than a hypoblastic structure (possibly of a germ origin). Sarcoma is easier to affect with treatment than carcinoma; a mole is more amenable to treatment than a wart.

However, any treatment which is palliative in inoperable sarcoma must be accepted as good, and we can only hope that the *vis medicatrix naturæ* and these palliative treatments may yet accomplish more than the pathological characters of the disease would lead us to expect. It is doubtful if cataphoresis carried the drug to a sufficient depth for any beneficial effect.)

---

TREATMENT OF ECZEMA BY PICRIC ACID. Gaucher. (*Soc. Méd. des Hôpitaux,* May 21, 1897; *La Presse Médicale,* No. 42, 1897, p. ccxxi.)

In cases of acute eczema, and in acute exacerbations of the same disease, the author painted on a one per cent. solution of picric acid, and then applied a layer of wool or butter-cloth soaked in the solution. The dressings were left on for two days. The inflammatory symptoms had then to a great extent subsided. The application was repeated every second day, and in a short time the acute inflammatory symptoms had disappeared. Gaucher found that the picric acid rapidly allayed irritation. According to the author, the treatment is only of use in vesicular, weeping eczema. He thinks the same treatment might be used in other superficial moist inflammations of the skin, and especially in all forms of pemphigus.—*George Pernet. British Journal Dermatology, August, 1897.*

---

VARICELLOUS STAPHYLOCOCCIA. Pirro Bolognini. (*La Pediatria,* 1897, No. 3, p. 76.—Abstract by Feindel in *La Presse Médicale,* No. 37, 1897, p. 204)

The author gives the details of a limited epidemic of varicella, in which nearly all the cases (twelve out of fifteen) terminated by a complication rare in itself, and which, in the epidemic in point, was exceptionally constant. In all the children in whom it was observed, the attack of varicella followed its usual course up to the time of the drying up of the vesicles, when, instead of drying up, they rapidly increased in size. In the course of twenty-four hours they had reached the stage of bullæ, the size of a two centime piece. A few hours later these bullæ burst, the fluid contents being whitish and rather thick, and containing on examination numerous staphylococci and a few streptococci. In the only case that terminated fatally (abscess of kidney), the cultivations were purely streptococcal. In all the cases there was albumin in the urine, but there was no other evidence of nephritis. The albumin disappeared when desquamation occurred. In conclusion:—1. Staphylo-streptococcia may be a complication of varicella (*Hulot, Hutinel and Labbe, &c.*); 2. It may occur in nearly all the cases of one epidemic of varicella; 3. Prognosis must be guarded. It only becomes grave when there are visceral complications.—*George Pernet. British Journal of Dermatology, August, 1897.*

